# Maternal racism experience and cultural identity in relation to offspring telomere length

**DOI:** 10.1038/s41598-023-37555-6

**Published:** 2023-06-28

**Authors:** Zaneta Thayer, Laia Becares, Emma Marks, Kien Ly, Caroline Walker

**Affiliations:** 1 Department of Anthropology, Dartmouth College, Hanover, NH USA; 2 Department of Global Health and Social Medicine, King’s College London, London, UK; 3 Centre for Longitudinal Research–He Arak i Mua, University of Auckland, Auckland, New Zealand

**Keywords:** Psychology, Biomarkers, Risk factors

## Abstract

Racism is a determinant of individual and offspring health. Accelerated telomere shortening, an indicator of cellular aging, is a potential mechanism through which parental experience of racism could affect offspring. Here we longitudinally evaluated the relationship between maternal lifetime experience of an ethnically-motivated verbal or physical attack, as reported in pregnancy, with offspring telomere length in 4.5-year-old children. We also explored the potential association between positive feelings about one’s culture and offspring telomere length. Data come from a nationally representative, multi-ethnic birth cohort in Aotearoa New Zealand (NZ) (Māori N = 417, Pacific N = 364, Asian N = 381). In models adjusting for covariates, including socioeconomic status and health status, Māori mothers who experienced an ethnically-motivated physical attack had children with significantly shorter telomere length than children of Māori mothers who did not report an attack (B = − 0.20, p = 0.01). Conversely, Māori mothers who had positive feelings about their culture had offspring with significantly longer telomeres (B = 0.25, p = 0.02). Our results suggest that ethnicity-based health inequities are shaped by racism, with impacts for clinical care and policy. Future research should also evaluate the potential protective effects of positive cultural identity.

## Introduction

Racism can be defined as a system that “unfairly disadvantages some individuals and communities, and unfairly advantages others”^1^. Racism can be persistent and unpredictable, thus requiring constant vigilance^2^. These features led Arlene Geronimus to propose the “Weathering hypothesis,” which argued that African American women in the United States experience accelerated biological aging in response to persistent exposure to structural and interpersonal racism across the life course^3^. It has since become recognized that systems of oppression—including racism—are a determinant of health inequities^4^ and could explain excess morbidity and mortality among African American women and other socially disadvantaged groups globally^5–8^.

One way the Weathering hypothesis has been evaluated is by exploring the association between stressors, such as inter-personal discrimination experience, and telomere length^9^. Telomeres are the caps at the end of chromosomes and become progressively shorter with each mitotic division. Because of this, telomere length is considered a biomarker of cellular aging. In addition to shortening as a natural process of aging, studies have found that psychosocial stressors, including discrimination experience, accelerate the rate of telomere shortening^10^.

Notably, studies evaluating the relationship between racial discrimination experience and telomere length have exclusively occurred within the United States and have only considered discrimination and telomere length among African American, White, or Hispanic populations^11–19^. These studies have found that racial discrimination is associated with shorter telomere length in some instances, but these associations sometimes differ according to factors such as ethnicity, depression, internalized racism, socioeconomic status, and ability to speak about one’s experience with others^9^.

In addition to impacting the biology and health of the present generation, it is now recognized that experiences of stress and threat in one generation can impact biological functioning and health in subsequent generations^20–22^. Experience of stressful life events in pregnancy, for example, has been associated with shorter telomere length among young adult offspring^23^, potentially due to fetal programming of telomere length at birth. *However, the extent to which mothers’ racial discrimination experience may associate with telomere length in offspring has not been explored.*

Here we evaluate the association between maternal report of an ethnically-motivated physical or verbal attack, as measured in pregnancy, with subsequent telomere length in their four and a half-year-old children using a multiethnic birth cohort from Aotearoa NZ. Prior research has demonstrated that racism is associated with variation in health in this cultural context^8^. We focus on the experience of an ethnically-motivated physical or verbal attack since these are particularly stressful experiences of discrimination and therefore have a greater potential for intergenerational impacts. Since previous work has described the moderating effects of internalized racism^13^), as a secondary hypothesis we explored the main and potential interactive effects between discrimination experience and whether mothers had positive, neutral, or negative feelings about their culture. Given that our prior research has demonstrated ethnic differences in telomere length in this cohort^24^, as well as within-group effects of discrimination on birth outcomes^25^, we also evaluated the relationship between maternal discrimination experience and offspring telomere length separately for each of the non-European broad ethnic groups: Māori (the Indigenous population), Pacific, and Asian.

## Results

Summary sample characteristics are presented in Table 1. Average maternal age at the antenatal data collection wave was 28.8–30.3 years old, depending on maternal ethnicity, and mean household incomes for Māori and Asian families were $50,000–69,999 NZ, while for Pacific families it was $30,000–49,999NZ. Asian mothers tended to be older and to have higher levels of education, while Pacific mothers were most likely to live in a neighborhood in the highest quintile for area-level deprivation. Lifetime experience of an ethnically-motivated verbal attack was commonly reported, with 17.1% of Pacific, 23.3% of Asian, and 31.6% of Māori participants reporting these experiences. Lifetime experience of an ethnically-motivated physical attack was more common among Māori (4.9%) and Pacific (4.6%) mothers than among Asian mothers (1.1%). Most participants had positive feelings about their culture (Māori: 85.2%, Pacific: 88.7%; Asian: 89.3%).

**Table 1:** Summary of study sample characteristics.

	Māori (N = 392)	Pacific (N = 345)	Asian (N =365)
Independent variables			
Experienced verbal attack	124 (31.6%)	59 (17.1%)	85 (23.3%)
Experienced physical attack	19 (4.9%)	16 (4.6%)	suppressed
Positive feelings about your culture	334 (85.2%)	306 (88.7%)	326 (89.3%)
Dependent variable			
Child telomere length (Log T/S ratio)	0.31 (0.36)	0.43 (0.32)	0.33 (0.35)
Covariates			
Maternal age (years)	28.8 (6.1)	29.0 (6.2)	30.3 (4.5)
Household income band	4.2 (1.6)	3.8 (1.7)	4.3 (1.5)
Bachelor’s degree or higher	93 (23.7%)	49 (14.2%)	189 (51.8%)
Top quintile area deprivation	175 (44.6%)	222 (64.3%)	78 (21.4%)
Prenatal depression score	6.3 (5.0)	8.0 (5.5)	5.6 (5.0)
Perceived stress score	13.7 (6.5)	15.9 (6.3)	12.4 (6.4)
Child sex (male)	205 (52.3%)	176 (51.0%)	213 (58.4%)
Birth weight (grams)	3487.2 (615.0)	3664.2 (647.9)	3277.9 (559.1)
Gestation length (weeks)	39.1 (2.0)	39.0 (2.0)	39.0 (1.8)
Maternal smoker	169 (43.1%)	105 (30.4%)	23 (6.3%)

Mean (SD) reported for continuous variable, N (%) for categorical variable. Cells with $<10$ observations are suppressed.

**Table 2:** Regression estimates and 95% confidence intervals from models evaluating the relationship between reporting an ethnically-motivated physical attack and log T/S ratios while adjusting for covariates.

Characteristic	Māori			Pacific			Asian		
	Beta	95% CI	p-value	Beta	95% CI	p-value	Beta	95% CI	p-value
Physical attack									
No physical attack	Ref	–		Ref	–		Ref	–	
Physical attack	− 0.20	− 0.34, − 0.05	**0.010**	− 0.08	− 0.24, 0.08	0.3	− 0.02	− 0.31, 0.26	0.9
Feelings about your culture									
Positive	Ref	–		Ref	–		Ref	–	
Neither positive nor negative	− 0.10	− 0.19, 0.00	**0.042**	− 0.02	− 0.13, 0.10	0.8	0.04	− 0.07, 0.15	0.5
Negative	− 0.25	− 0.46, − 0.04	**0.020**	− 0.18	− 0.40, 0.05	0.13	− 0.17	− 0.61, 0.28	0.5
Prenatal depression	0.05	− 0.06, 0.16	0.4	− 0.07	− 0.16, 0.02	0.13	0.00	− 0.12, 0.11	> 0.9
Maternal age	0.01	0.00, 0.01	**0.013**	0.01	0.00, 0.01	**0.007**	0.00	− 0.01, 0.01	0.8
Household income	− 0.01	− 0.03, 0.01	0.3	− 0.01	− 0.03, 0.01	0.2	0.00	− 0.03, 0.02	0.7
Education	− 0.01	− 0.09, 0.07	0.9	0.01	− 0.09, 0.10	> 0.9	0.03	− 0.04, 0.10	0.4
Top quintile area deprivation	− 0.03	− 0.10, 0.03	0.3	0.07	0.00, 0.14	0.066	− 0.01	− 0.09, 0.07	0.8
Female offspring sex	0.06	− 0.01, 0.12	0.083	0.06	− 0.01, 0.13	0.078	0.08	0.01, 0.14	**0.022**
Offspring birth weight (grams)	0.00	0.00, 0.00	0.4	0.00	0.00, 0.00	0.4	0.00	0.00, 0.00	**0.018**
Gestational age at birth (days)	− 0.01	− 0.03, 0.01	0.5	− 0.01	− 0.03, 0.01	0.3	0.01	− 0.01, 0.03	0.5
Perceived stress score	0.00	0.00, 0.01	0.6	0.00	0.00, 0.01	0.2	− 0.01	− 0.01, 0.00	**0.047**
Maternal smoker	0.01	− 0.06, 0.08	0.7	0.02	− 0.05, 0.10	0.6	0.09	− 0.05, 0.23	0.2
McFadden’s psuedo R^2^			0.12			0.13			0.11

P-values $< 0.05$ are in bold.

Greater maternal age was associated with significantly longer offspring telomere length in several but not all models (Tables 2, 3), and female offspring tended to have longer telomeres than male offspring. Perceived stress score measured in pregnancy was associated with significantly shorter telomere length in offspring, but only among children of Asian mothers. Maternal depression, perceived stress, income, education, area deprivation, smoking in pregnancy, and gestational age at birth were not significantly associated with offspring telomere length in any of the models.

**Figure 1:**
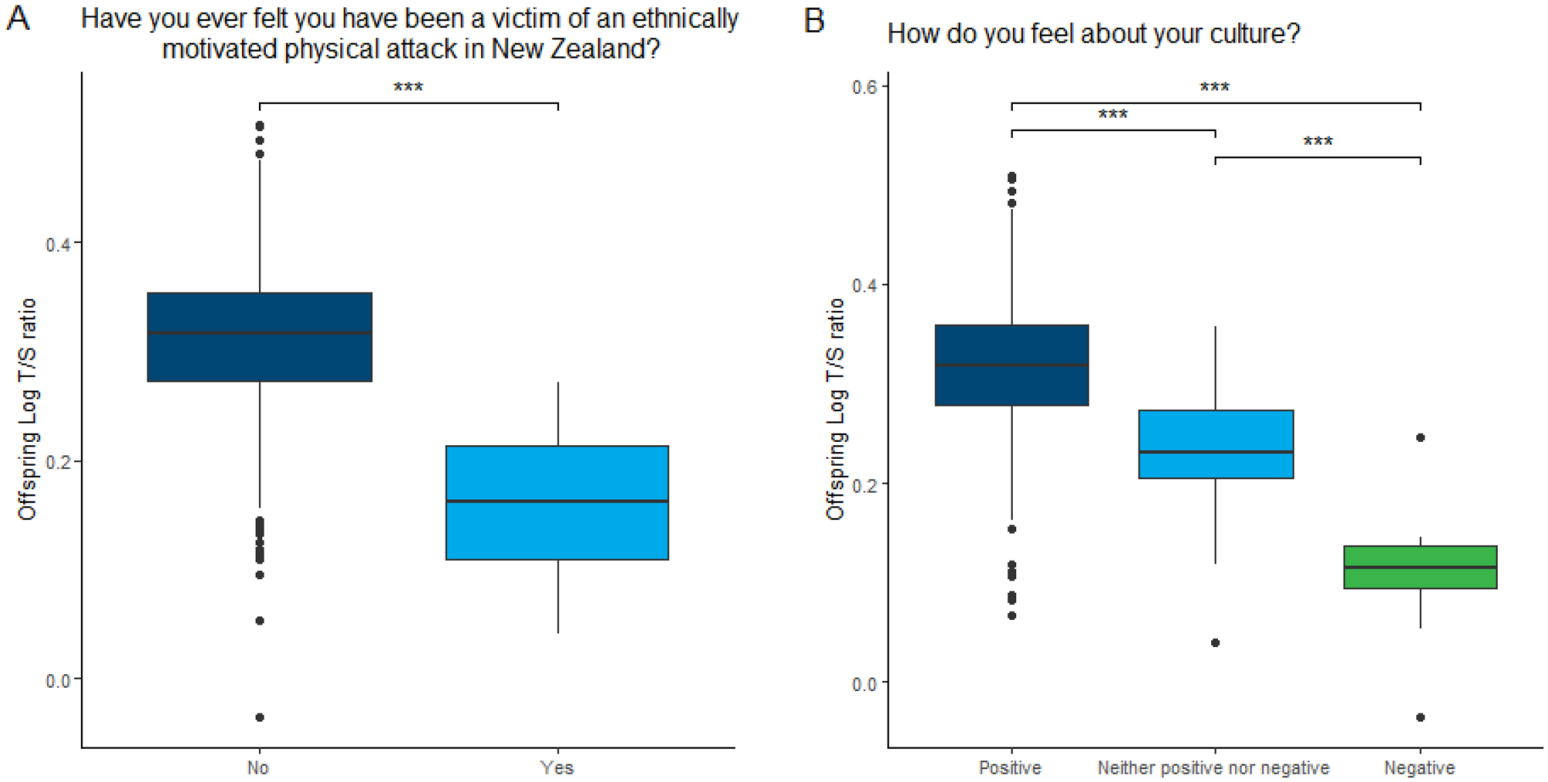
(**A**) Children of Māori mothers who reported experiencing an ethnically motivated physical attack have significantly shorter telomere length than children of Māori mothers not reporting a similar experience. (**B**) Children of Māori mothers who felt positively about their culture have longer telomeres than children of Māori mothers who felt either negative or neither positive nor negative feelings about their culture.

Māori mothers who reported experiencing a physical attack had children with significantly shorter telomeres than children of Māori mothers who did not similarly experience a physical attack (B = − 0.20, p = 0.01) (Fig. 1A). Although the coefficients were negative for all of the ethnically-motivated attack variables in relation to telomere length, none of the other analyses, including those in children of Pacific and Asian mothers, reached statistical significance (Tables 2, 3).

In both the physical and verbal ethnically-motivated attack models, a positive feeling about one’s culture was associated with significantly longer telomeres among children of Māori mothers when compared to both those with a negative feeling about their culture (physical attack model: B = − 0.25, p = 0.020) and those with neither a positive nor negative feeling (physical attack model: B = − 0.10, p = 0.04) (Fig. 1B). There was no significant interaction between the discrimination measures and feelings about one’s culture (data not shown).

## Discussion

Racism, as commonly indexed by interpersonal discrimination experience, has been consistently associated with increased morbidity and mortality^4^. Experience of discrimination has also been associated with increased cellular aging, as measured by telomere length, but only within the United States and, among Hispanic, White and/or African Americans. In addition, while racial discrimination experienced by mothers has been associated with offspring phenotypes including birth size and stress reactivity^25–27^, no prior work has evaluated the intergenerational impact of discrimination experience on telomere length.

Here, we evaluated whether maternal experience of discrimination and feelings about one’s culture, as measured during pregnancy, were associated with telomere length in offspring measured in early childhood (4.5 years). We found that children of Māori women who experienced an ethnically-motivated physical attack had significantly shorter telomere length in childhood, compared to children of Māori women who had not experienced an ethnically-motivated physical attack. We also found that positive feelings about one’s culture was associated with significantly longer telomeres among children of Māori mothers. The effect sizes for both variables were four to six times greater than the next largest coefficient (offspring sex), suggesting a biologically significant effect. Differences in telomere length that emerge in childhood may be maintained into adulthood, therefore contributing to the accumulation of health inequity across the life span. In support of this hypothesis, a longitudinal study of parents and children that collected telomere length at baseline and 14 years later found a similar correlation in measurement across time in parents, who were an average of 43 years at baseline, with their children who were an average of 11 at baseline. This suggests that inter-individual differences in telomere length are influenced by environmental factors prior to age 11^28^.

**Table 3:** Regression estimates and 95% confidence intervals from models evaluating the relationship between reporting an ethnically-motivated verbal attack and log T/S ratios while adjusting for covariates.

Characteristic	Māori			Pacific			Asian		
	Beta	95% CI1	p-value	Beta	95% CI1	p-value	Beta	95% CI1	p-value
Verbal attack									
No verbal attack	Ref	–		Ref	–		Ref	–	
Verbal attack	− 0.04	− 0.11, 0.03	0.2	− 0.05	− 0.14, 0.04	0.2	0.00	− 0.08, 0.07	> 0.9
Feelings about your culture									
Positive	Ref	–		Ref	–		Ref	–	
Neither positive nor negative	− 0.10	− 0.19, 0.00	**0.042**	− 0.01	− 0.12, 0.11	> 0.9	0.04	− 0.07, 0.15	0.5
Negative	− 0.24	− 0.45, − 0.03	**0.025**	− 0.20	− 0.42, 0.03	0.084	− 0.17	− 0.61, 0.28	0.5
Prenatal depression	0.04	− 0.07, 0.15	0.5	− 0.01	− 0.01, 0.00	0.14	0.00	− 0.12, 0.11	> 0.9
Maternal age	0.01	0.00, 0.01	**0.034**	0.01	0.00, 0.01	**0.004**	0.00	− 0.01, 0.01	0.8
Household income	− 0.01	− 0.03, 0.01	0.4	− 0.01	− 0.03, 0.01	0.2	0.00	− 0.03, 0.02	0.7
Education	0.00	− 0.08, 0.08	> 0.9	0.01	− 0.09, 0.11	0.9	0.03	− 0.04, 0.10	0.4
Top quintile area deprivation	− 0.04	− 0.10, 0.03	0.2	0.06	− 0.01, 0.13	0.084	− 0.01	− 0.09, 0.07	0.8
Female offspring sex	0.06	0.00, 0.12	0.062	0.06	− 0.01, 0.12	0.10	0.08	0.01, 0.15	**0.021**
Offspring birth weight (grams)	0.00	0.00, 0.00	0.4	0.00	0.00, 0.00	0.5	0.00	0.00, 0.00	**0.018**
Gestational age at birth (days)	− 0.01	− 0.02, 0.01	0.6	− 0.01	− 0.03, 0.01	0.4	0.01	− 0.01, 0.03	0.5
Perceived Stress Score	0.00	0.00, 0.01	0.7	0.01	0.00, 0.01	0.12	− 0.01	− 0.01, 0.00	**0.046**
Maternal smoker	0.01	− 0.06, 0.08	0.8	0.02	− 0.05, 0.10	0.5	0.09	− 0.05, 0.22	0.2
McFadden psuedo R^2^			0.10			0.13			0.11

P-values $< 0.05$ are in bold.

These results are consistent with our prior work reporting that discrimination experience of mothers is associated with offspring phenotype among Māori but not non-Māori women. Specifically, we have previously reported that discrimination was associated with reduced birth weight and shorter gestation length in the same sample^25^. The significant result in our present analysis remains even with adjustment for birth weight and gestation length, suggesting that the effects of discrimination on telomere length were independent of the impacts of ethnic discrimination on birth outcomes previously reported.

It is important to note that since our model adjusts for factors that are patterned by racism that could also affect stress and telomere length (e.g., education, income, and deprivation area), these estimates potentially underestimate the total impacts of racism on telomere length. For example, a US study found that living in a high deprivation neighborhood, which in Aotearoa NZ is more common for Māori and Pacific children and is shaped by institutional racism^29,30^, is associated with shortened telomere length among adults^31^. Racism therefore impacts individuals lived experiences beyond those reflected here in racially motivated physical and verbal attacks. Nonetheless, these findings highlight and build on previous work demonstrating that racism contributes to well-documented health inequities between Māori and non-Māori within Aotearoa NZ^32^.

These results are also broadly consistent with work from the US in multi-ethnic cohorts that have reported significant associations between discrimination experience and telomere length in some ethnicities and sub-groups but not others^11,15,17^. Differences in findings could relate to some studies being underpowered to detect significant effects when comparing within ethnicities or could reflect structural differences among socially defined racial groups that shape the amount and nature of discrimination experience. For example, a community study in Nashville, Tennessee reported that unfair treatment by the police predicted shorter telomere length among Black but not White men^16^. Another study reported an increase in pregnancy loss for Black, but not White, individuals who were locally exposed to fatal police violence during gestation^33^. In sum, the psychological, and therefore the physiological, effect of discrimination experience likely varies according to individual history of discrimination experience and the sociocultural context within which that discrimination is occurring.

Previous research has demonstrated that discrimination has adverse health effects for groups that encounter systematic bias, including indigenous Māori. However, less research has identified factors that may buffer against these associations. Positive cultural identity may reduce the impacts of discrimination experienced through awareness of strengths, resources, and values related to one’s culture^34^. Consistent with this, Māori cultural identity has been described as a “critical prerequisite” for wellness^35^. Previous research among rangatahi (young people) Māori reported that strong cultural identity was associated with better mental health outcomes, even in models adjusting for discrimination experience^36^. We found that, among Māori participants, positive feelings about one’s culture was associated with longer offspring telomere length. These findings suggest that interventions that promote individual connection with whānau, iwi, hapū, and Tikanga Māori can strengthen cultural identity and potentially improve outcomes^37^. That said, clinicians and policymakers must also enact policies to dismantle the structural racism that create racial inequities in the first place^38^. In Aotearoa NZ specifically, this could include supporting changes to constitutional arrangements to better support Māori^39^. For clinicians, this includes using a race-conscious as opposed to race-based approach to medicine^40^ and conveying openness to discussing racism without forcing undesired conversations^41^.

The data from this study come from a longitudinal, nationally representative sample. That said, there are important limitations to the research. First, we are focusing here on a very limited scope of racism; that of verbal or physical attacks attributed to participants’ race. Systemic racism can take many forms, including discrimination in seeking housing, medical care, education, and employment, residential segregation, and access to resources to promote health. The association between explicit forms of racist attacks and offspring telomere length reported here should therefore be interpreted as a minimum estimate for the overall influence of racism. Second, we do not ask about the specific number or timing of lifetime racist attacks; differences in the amount or recentness of the attack could be important and should be assessed in future research.

In sum, we found that Māori women who experienced an ethnically-motivated physical attack during or prior to pregnancy had children with significantly shorter telomere length measured at 4.5 years of age when compared to the children of Māori mothers who did not report an ethnically-motivated physical attack. We also found that Māori mothers with a positive cultural identity had offspring with significantly longer telomeres. We encourage interventions that both dismantle racism and promote cultural connection.

## Methods

### Cohort

This study utilizes the Growing Up in New Zealand (GUiNZ) longitudinal study of child development. The only inclusion criteria were having an estimated delivery date between 25 April 2009 and 25 March 2010 and being resident, while pregnant, within the broad geographical area of three District Health Board regions (Auckland, Counties-Manukau and Waikato) in Aotearoa NZ’s North Island. This region was chosen because of the ability to sample a birth cohort that would be broadly applicable to the sociodemographic diversity of contemporary births in Aotearoa NZ. A record of all births in the country between 2003 and 2007 was used as a reference population to ensure the multiple recruitment strategies used led to an appropriately diverse cohort. There were no exclusion criteria^42^. The resulting birth cohort of 6,853 children includes 33% of the births within the geographical area sampled, and 11% of the births in the entire country during the recruitment period^42^. For the antenatal data collection, participants were visited by interviewers at their homes predominantly during the third trimester of pregnancy to complete a face-to-face computer-assisted interview.

Ninety percent of participants (N = 6156) completed the 4-year data collection wave, which occurred using the same methods as the antenatal data collection. Of those children completing the 4-year data collection wave, 25% (N = 1522) were identified as Māori, 21% (N = 1263) were identified as Pacific and 18% (N = 1027) were identified as Asian. Ethical approval for the study was obtained from the Ministry of Health Northern Y Regional Ethics Committee (Ref NTY/08/06/055). All research was performed in accordance with relevant guidelines and regulations. Written informed consent was obtained from all participating women.

### Dependent variable: telomere length

#### Telomere sample collection

Saliva samples were obtained from the participants during the face-to-face interview conducted when the children were 54 months of age using the Oragene DNA saliva Self-Collection kit (OG-575, DNA GenoTek Inc.), with samples stored at room temperature until processing. DNA extractions were carried out based on the manufacturer’s protocol. The extracted DNA samples were re-suspended in TE (10 mM Tris, 1 mM EDTA, pH $8\cdot 0$) and stored at $-80\,^{\circ }\text {C}$. Telomere measurement data at 54 months were available for 4394 of the 6853 children participating in GUiNZ (Māori N = 598; Pacific N = 564; Asian N = 536).

#### Measurement of relative telomere length

Relative telomere length were determined by quantitative PCR based on a previously published protocol as described previously^24^. The relative telomere length for each sample was expressed in T/S (telomere over single copy number gene) ratio and was calculated using the − $\Delta \Delta \hbox {Ct}$ formula with efficiency taken into account:

$$\begin{aligned} T/S = \frac{E_{tel}^{(C_t tel - standard - C_t tel-sample)}}{E_{alb}^{(C_t alb-standard - C_t alb-sample)} } \end{aligned}$$

where Etel and Ealb are the amplification efficiency of the telomere and albumin sequence respectively, Cttel-standard and Cttel-sample are the Ct for the telomere sequence of the standard DNA and that of the sample respectively, Ctalb-standard and Ctalb-sample are the Ct for the telomere sequence of the standard DNA and that of the sample respectively. The T/S ratio is expected to be proportional to the average telomere length per cell. A T/S ratio $>1$ indicates that the measured sample has an average telomere length greater than that of standard DNA. Conversely, a T/S ratio $<1$ indicates that the sample has an average telomere length shorter than that of standard DNA. T/S ratios were natural log transformed to achieve normality before we undertook further analyses.

### Indpendent variables

#### Experience of an ethnically-motivated attack

In the antenatal questionnaire, women were asked a series of questions regarding lifetime and past year experiences of ethnic discrimination^42^. This included physical attacks and verbal attacks that individuals attributed to their ethnicity. For all questions participants were able to answer ‘yes, within the past 12 months;’ ‘Yes, more than 12 months ago;’ and ‘No.’ We analyzed whether individuals had ever experienced either physical and/or verbal attack (yes/no for physical attack or verbal attack, respectively, in any period).

#### Feeling about one’s culture

In the antenatal questionnaire, women were asked, “How do you feel about your culture?” and were able to answer: (1) Very positive, (2) Fairly positive, (3) Neither positive nor negative, (4) Slightly negative, or (5) Very Negative. The variable was collapsed to “Positive” (1-2), “Neither positive nor negative” (3) or “Negative” (4-5) for analysis.

### Covariates

#### Maternal mental health

Edinburgh Depression Survey, considered the gold standard for measurement of depression during the perinatal period. An Edinburgh depression score ≥ 13 was considered an indicator of probable depression^43^. The Perceived Stress survey was administered during the antenatal questionnaire to assess perceived stress^44^.

#### Socio-demographic variables

Maternal ethnic identification was described during the antenatal interview and was classified as Māori, Pacific (including individuals identified as Samoan, Tongan, Cook Islands Māori, Niuean, or Other Pacific Peoples), European, Asian or Other^45^. Maternal age (years), household income, and educational attainment were self-reported at the antenatal interview. Education was measured as (0 = Trade certification/National Certificate Levels 1-4; 1 = Diploma below bachelor or National Certificate 5 or 6; 2 = Bachelor’s Degree; 3 = Bachelor’s degree with honors or postgraduate diploma; 4 = Higher degree) and re-classified as Bachelor’s degree with honors or higher degree as opposed to those with a Bachelor’s degree or less. Yearly household income was collected as (1 = $\le$ 20,000 New Zealand Dollars (NZD); 2 = 20,000–29,999NZD; 3 = 30,000–49,999NZD; 4 = 50,000–69,999NZD; 5 = 70,000–99,999NZD; 6 =100,000–149,999NZD; 7 $\ge$ 150,000NZD) during the antenatal survey. Area level deprivation (1–10, with 10 being most deprived) was calculated based on address information from the antenatal interview^46^, with participants classified as living in a high deprivation area if they had an Area Deprivation Score $\ge$ 9.

#### Other covariates

Maternal smoking was collected during the antenatal survey and the 9-month postnatal survey. Since offspring telomere length has been previously associated with pre- and post-conception maternal smoking, individuals who reported smoking regularly before they were aware they were pregnant (20.0% of sample), who were smoking at the time of the antenatal interview (10.5% of sample), or who were smoking at the time of the 9-month interview (13.9%) were categorized as a smoker. Birth outcome information (offspring birth weight, gestation length) was collected from linkage to medical health records.

### Statistical analysis

Descriptive statistics were generated for all study variables according to maternal ethnicity (Māori, Pacific, Asian). Multivariate regression was used to assess the study hypotheses. Models were constructed to evaluate the relationship between telomere length and verbal and physical attack, respectively, separately for each ethnicity. These models adjusted for maternal age (continuous), education (continuous), household income (continuous), high area deprivation (dichotomous), prenatal depression score (dichotomous), perceived stress score (continuous), feeling about one’s culture (categorical), smoking during pregnancy (dichotomous), offspring sex (dichotomous), gestational age at birth (continuous) and birth weight (continuous). We then explored if there were significant interactions between each of the discrimination variables and feeling about one’s culture. VIFS were in acceptable ranges for all models (1.04–1.79). P-values less than 0.05 were considered statistically significant. Statistical analyses and data visualizations were performed using R.

## Data Availability

The data that support the findings of this study are available from the Growing Up In New Zealand Data Access Committee. However, restrictions apply to the availability of these data, which were used under license for the current study and are not publicly available. Data are available from the Growing Up In New Zealand Data Access Committee with their permission (dataaccess@growingup.co.nz).
